# *catena*-Poly[sodium-μ_2_-(*N*,*N*,*N*′,*N*′-tetra­methyl­ethane-1,2-diamine)-κ^2^ *N*:*N*′-sodium-bis­[μ_2_-bis­(trimethyl­sil­yl)aza­nido-κ^2^ *N*:*N*]]

**DOI:** 10.1107/S1600536812045126

**Published:** 2012-11-10

**Authors:** Alan R. Kennedy, Robert E. Mulvey, Charles T. O’Hara, Stuart D. Robertson, Gemma M. Robertson

**Affiliations:** a WestCHEM, Department of Pure and Applied Chemistry, University of Strathclyde, 295 Cathedral Street, Glasgow G1 1XL, Scotland

## Abstract

The title compound, [Na_2_(C_6_H_18_NSi_2_)_2_(C_6_H_16_N_2_)]_*n*_, was found to consist of dimeric [Na(NSiMe_3_)_2_] units with crystallographically imposed centrosymmetry based upon four-membered NaNNaN rings. The dimers are bridged by *N*,*N*,*N*′,*N*′-tetra­methyl­ethylenediamine ligands, which act in an unusual extended non-chelating coordination mode. This gives a one-dimensional coordination polymer that extends parallel to the *a*-axis direction.

## Related literature

For structures of non-solvated [Na(NSiMe_3_)_2_], see: Grüning & Atwood (1977 ▶); Driess *et al.* (1997 ▶); Knizek *et al.* (1997 ▶) and for THF-solvated [Na(NSiMe_3_)_2_], see: Sarazin *et al.* (2006 ▶); Karl *et al.* (1999 ▶). For similar complexes with diamine bridges between metal atoms, see: Henderson *et al.* (1997 ▶); Bernstein *et al.* (1992 ▶).
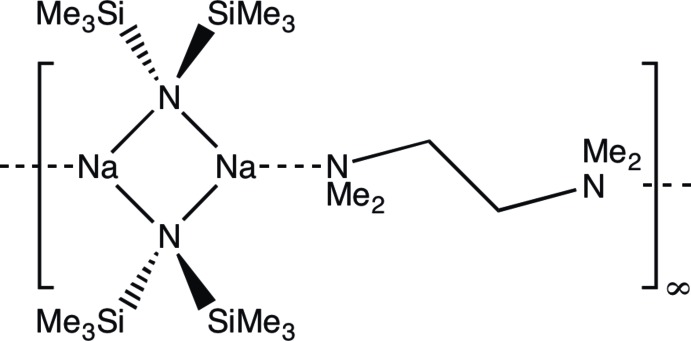


## Experimental

### 

#### Crystal data

[Na_2_(C_6_H_18_NSi_2_)_2_(C_6_H_16_N_2_)]
*M* *_r_* = 482.98
Monoclinic, 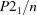

*a* = 9.9761 (12) Å
*b* = 13.8292 (12) Å
*c* = 11.7983 (14) Å
β = 113.523 (14)°
*V* = 1492.4 (3) Å^3^
*Z* = 2
Mo *K*α radiation
μ = 0.24 mm^−1^
*T* = 123 K
0.20 × 0.14 × 0.10 mm

#### Data collection

Oxford Diffraction Gemini S diffractometer
Absorption correction: multi-scan (*CrysAlis PRO*; Oxford Diffraction, 2009 ▶) *T*_min_ = 0.942, *T*_max_ = 1.000
8332 measured reflections
3436 independent reflections
3064 reflections with *I* > 2σ(*I*)
*R*_int_ = 0.012

#### Refinement

*R*[*F* ^2^ > 2σ(*F* ^2^)] = 0.023
*wR*(*F* ^2^) = 0.067
*S* = 1.07
3436 reflections
135 parameters
H-atom parameters constrained
Δρ_max_ = 0.37 e Å^−3^
Δρ_min_ = −0.18 e Å^−3^

### 

Data collection: *CrysAlis PRO* (Oxford Diffraction, 2010) ▶; cell refinement: *CrysAlis PRO*; data reduction: *CrysAlis PRO*; program(s) used to solve structure: *SHELXS97* (Sheldrick, 2008 ▶); program(s) used to refine structure: *SHELXL97* (Sheldrick, 2008 ▶); molecular graphics: *DIAMOND* (Brandenburg, 1999 ▶); software used to prepare material for publication: *SHELXL97*.

## Supplementary Material

Crystal structure: contains datablock(s) I, global. DOI: 10.1107/S1600536812045126/pk2451sup1.cif

Structure factors: contains datablock(s) I. DOI: 10.1107/S1600536812045126/pk2451Isup2.hkl

Additional supplementary materials:  crystallographic information; 3D view; checkCIF report

## Figures and Tables

**Fig. 1.**
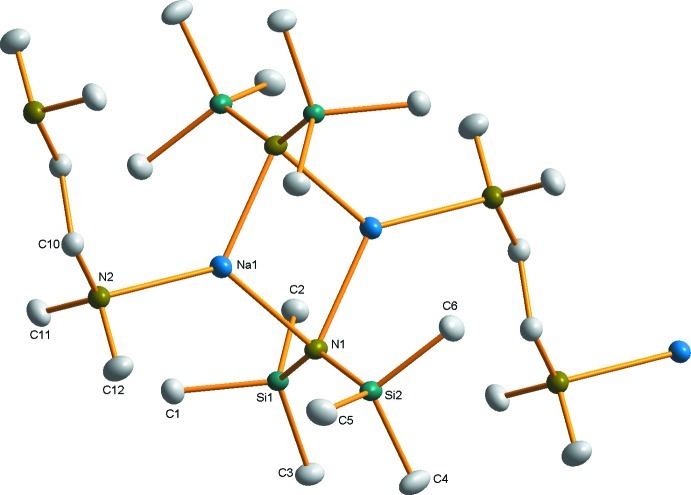
View of the central dimeric unit of (I) showing 50% probability displacement ellipsoids.

**Fig. 2.**
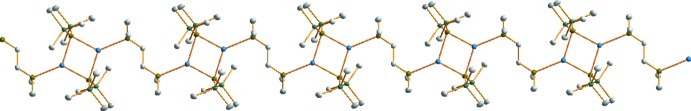
Section of the one-dimensional polymeric coordination chain of (I) that extends along the crystallographic a direction.

## supplementary crystallographic information

### Comment

Sodium 1,1,1,3,3,3-hexamethyldisilazide [Na(NSiMe_3_)_2_] is particularly useful in synthetic chemistry for the deprotonation reaction (the change of an inert C—H bond into a more useful C-metal bond) due to its low nucleophilicity and strong Brønsted basicity. The reactivity of this and similar organometallic reagents can generally be enhanced by the presence of a Lewis donor which operates by ligating the Lewis acidic metal centre and giving a lesser degree of oligomerization. One of the most common donors for this purpose is the diamine *N,N,N',N'*-tetramethylethylenediamine (TMEDA) which typically binds in a bidentate fashion. The unsolvated parent sodium amide is known to crystallize as either a one-dimensional chain polymer (Grüning & Atwood, 1977) or as a cyclotrimer (Knizek *et al.*, 1997; Driess *et al.*, 1997). Cyclodimeric Na(NSiMe_3_)_2_ ring forms with either one (Sarazin *et al.*, 2006) or both Na centres solvated by tetrahydrofuran (Karl *et al.*, 1999) are also known. We now report the structure of [Na(NSiMe_3_)_2_]_2_.(TMEDA), (I), a Na_2_N_2_ cyclodimer with TMEDA binding to two sodium centres in a relatively unusual bridging rather than chelating fashion. A similar Li_2_N_2_ cyclodimer (N belonging to the related utility amide diisopropylamide, N*^i^*Pr_2_) bridged by TMEDA ligands (Bernstein *et al.*, 1992) and a sodium hexamethyldisilazide cyclodimer bridged by the related ligand TMPDA, Me_2_N(CH_2_)_3_NMe_2_, (Henderson *et al.*, 1997) have previously been reported.

(I) was prepared by mixing NaN(SiMe_3_)_2_ and TMEDA in a 2:1 ratio in hexane. The core structural motif is a crystallographically centrosymmetric four membered ring consisting of two Na centres and two N atoms from hexamethyldisilazide anions, with TMEDA binding to Na in a monodentate fashion, see Fig. 1. By acting as a bridging ligand to another Na_2_N_2_ ring, the TMEDA propagates the structure as a "polymer of dimers". This one dimensional polymer runs along the crystallographic *a* direction, see Fig. 2. The Na centres are three-coordinate and distorted trigonal planar. The Na—N_HMDS_ distances [2.436 (1) and 2.451 (1) Å] are consistent with those found in the related polymer of dimers with TMPDA acting as the bridging ligand (Henderson, *et al.* 1997), the Na—N_donor_ bond lengths are also comparable between these two structures.

### Experimental

All experimental manipulations were performed under an atmosphere of argon using either standard Schlenk techniques or a MBraun glove box fitted with an inert gas recirculation and purification system. NaHMDS was purchased from Aldrich and used as received. Hexane and toluene were distilled over sodium benzophenone prior to use. TMEDA was distilled over CaH_2_ and stored over 4 Å molecular sieves prior to use. NMR spectroscopy data were recorded on a Bruker AV400 MHz spectrometer operating at 400.13 MHz for ^1^H and 100.62 MHz for ^13^C. Na(NSiMe_3_)_2_ (0.73 g, 4 mmol) was suspended in dried hexane (10 ml) in an oven-dried Schlenk flask and this was sonicated for 10 min to give a white suspension. TMEDA (0.30 ml, 2 mmol) was introduced, followed by toluene (7.5 ml) to give a clear pale yellow solution. This was stirred for 3 h, heated and left in a freezer at 245 K. After 5 days only a small amount of crystalline material was observed. All solvents were removed under reduced pressure and toluene (8 ml) was added to give a clear yellow solution. This was heated and left to slowly cool, depositing a crop of colourless X-ray quality crystals in an 84% yield (0.81 g). ^1^H NMR (400 MHz, C_6_D_6_, 300 K): δ 1.99 (s, 4H, TMEDA CH_2_), 1.97 (s, 12H, TMEDA CH_3_), 0.19 (s, 36H, SiMe_3_). ^13^C NMR (100 MHz, C_6_D_6_, 300 K): δ 57.6 (TMEDA CH_2_), 45.8 (TMEDA CH_3_), 7.0 (SiMe_3_).

### Refinement

All H atoms were placed in idealized positions and refined in riding modes with C—H = 0.99 and 0.98 Å for CH_2_ and CH_3_ groups respectively and with *U*_iso_(H) = 1.2 or 1.5 times *U*_eq_(C) of the parent atom for CH_2_ and CH_3_ groups respectively. The orientation of the CH_3_ groups was refined by allowing rotation around the C—C and Si—C bonds.

### Crystal data

**Table:**

[Na_2_(C_6_H_18_NSi_2_)_2_(C_6_H_16_N_2_)]	*F*(000) = 532
*M**_r_* = 482.98	*D*_x_ = 1.075 Mg m^−^^3^
Monoclinic, *P*2_1_/*n*	Mo *K*α radiation, λ = 0.71073 Å
Hall symbol: -P 2yn	Cell parameters from 4914 reflections
*a* = 9.9761 (12) Å	θ = 2.9–29.1°
*b* = 13.8292 (12) Å	µ = 0.24 mm^−^^1^
*c* = 11.7983 (14) Å	*T* = 123 K
β = 113.523 (14)°	Block, colourless
*V* = 1492.4 (3) Å^3^	0.20 × 0.14 × 0.10 mm
*Z* = 2	

### Data collection

**Table:**

Oxford Diffraction Gemini S diffractometer	3436 independent reflections
Radiation source: fine-focus sealed tube	3064 reflections with *I* > 2σ(*I*)
Graphite monochromator	*R*_int_ = 0.012
ω scans	θ_max_ = 29.1°, θ_min_ = 3.0°
Absorption correction: multi-scan (*CrysAlis PRO*; Oxford Diffraction, 2009)	*h* = −13→13
*T*_min_ = 0.942, *T*_max_ = 1.000	*k* = −18→18
8332 measured reflections	*l* = −16→16

### Refinement

**Table:**

Refinement on *F*^2^	Primary atom site location: structure-invariant direct methods
Least-squares matrix: full	Secondary atom site location: difference Fourier map
*R*[*F*^2^ > 2σ(*F*^2^)] = 0.023	Hydrogen site location: inferred from neighbouring sites
*wR*(*F*^2^) = 0.067	H-atom parameters constrained
*S* = 1.07	*w* = 1/[σ^2^(*F*_o_^2^) + (0.0371*P*)^2^ + 0.2964*P*] where *P* = (*F*_o_^2^ + 2*F*_c_^2^)/3
3436 reflections	(Δ/σ)_max_ = 0.001
135 parameters	Δρ_max_ = 0.37 e Å^−^^3^
0 restraints	Δρ_min_ = −0.18 e Å^−^^3^

### Special details

**Table:**

Experimental. Absorption correction: CrysAlisPro, Oxford Diffraction Ltd., Empirical absorption correction using spherical harmonics, implemented in SCALE3 ABSPACK scaling algorithm.
Geometry. All e.s.d.'s (except the e.s.d. in the dihedral angle between two l.s. planes) are estimated using the full covariance matrix. The cell e.s.d.'s are taken into account individually in the estimation of e.s.d.'s in distances, angles and torsion angles; correlations between e.s.d.'s in cell parameters are only used when they are defined by crystal symmetry. An approximate (isotropic) treatment of cell e.s.d.'s is used for estimating e.s.d.'s involving l.s. planes.
Refinement. Refinement of *F*^2^ against ALL reflections. The weighted *R*-factor *wR* and goodness of fit *S* are based on *F*^2^, conventional *R*-factors *R* are based on *F*, with *F* set to zero for negative *F*^2^. The threshold expression of *F*^2^ > σ(*F*^2^) is used only for calculating *R*-factors(gt) *etc*. and is not relevant to the choice of reflections for refinement. *R*-factors based on *F*^2^ are statistically about twice as large as those based on *F*, and *R*- factors based on ALL data will be even larger.

### Fractional atomic coordinates and isotropic or equivalent isotropic displacement parameters (Å^2^)

**Table:**

	*x*	*y*	*z*	*U*_iso_*/*U*_eq_	
Si1	0.92808 (3)	0.192359 (19)	0.06098 (2)	0.01444 (8)	
Si2	1.02618 (3)	0.03467 (2)	0.26243 (2)	0.01598 (8)	
Na1	1.16231 (4)	0.03368 (3)	0.03282 (4)	0.01891 (10)	
N1	0.98789 (9)	0.08205 (6)	0.12110 (7)	0.01645 (17)	
N2	1.42648 (9)	0.08786 (6)	0.08534 (8)	0.01989 (18)	
C1	1.07176 (12)	0.26455 (8)	0.03194 (10)	0.0221 (2)	
H1A	1.0329	0.3287	0.0005	0.033*	
H1B	1.0986	0.2310	−0.0291	0.033*	
H1C	1.1584	0.2715	0.1094	0.033*	
C2	0.77062 (12)	0.17896 (8)	−0.09588 (10)	0.0215 (2)	
H2A	0.7373	0.2431	−0.1311	0.032*	
H2B	0.6899	0.1446	−0.0863	0.032*	
H2C	0.8034	0.1423	−0.1512	0.032*	
C3	0.86084 (12)	0.27808 (8)	0.15074 (10)	0.0220 (2)	
H3A	0.8189	0.3356	0.1006	0.033*	
H3B	0.9427	0.2973	0.2270	0.033*	
H3C	0.7859	0.2460	0.1714	0.033*	
C10	1.52737 (11)	0.01764 (8)	0.06673 (9)	0.0197 (2)	
H10A	1.5393	−0.0385	0.1220	0.024*	
H10B	1.6244	0.0482	0.0898	0.024*	
C4	0.95775 (14)	0.10439 (9)	0.36540 (10)	0.0282 (3)	
H4A	0.9842	0.0701	0.4440	0.042*	
H4B	0.8512	0.1106	0.3250	0.042*	
H4C	1.0022	0.1689	0.3809	0.042*	
C5	1.22784 (12)	0.01762 (9)	0.35863 (10)	0.0263 (2)	
H5A	1.2412	−0.0138	0.4368	0.039*	
H5B	1.2765	0.0808	0.3756	0.039*	
H5C	1.2704	−0.0229	0.3134	0.039*	
C11	1.42724 (12)	0.17946 (8)	0.02160 (11)	0.0250 (2)	
H11A	1.5278	0.2032	0.0488	0.038*	
H11B	1.3678	0.2275	0.0416	0.038*	
H11C	1.3866	0.1686	−0.0679	0.038*	
C6	0.94484 (13)	−0.09035 (8)	0.24995 (10)	0.0252 (2)	
H6A	0.9710	−0.1172	0.3329	0.038*	
H6B	0.9832	−0.1323	0.2029	0.038*	
H6C	0.8382	−0.0864	0.2075	0.038*	
C12	1.47846 (13)	0.10829 (10)	0.21849 (11)	0.0311 (3)	
H12A	1.5766	0.1366	0.2481	0.047*	
H12B	1.4818	0.0480	0.2632	0.047*	
H12C	1.4116	0.1538	0.2328	0.047*	

### Atomic displacement parameters (Å^2^)

**Table:**

	*U*^11^	*U*^22^	*U*^33^	*U*^12^	*U*^13^	*U*^23^
Si1	0.01536 (13)	0.01393 (14)	0.01336 (13)	0.00077 (10)	0.00503 (10)	−0.00045 (9)
Si2	0.02043 (15)	0.01380 (14)	0.01380 (14)	0.00041 (10)	0.00692 (11)	−0.00009 (9)
Na1	0.0168 (2)	0.0193 (2)	0.0217 (2)	−0.00234 (15)	0.00880 (17)	−0.00370 (15)
N1	0.0199 (4)	0.0148 (4)	0.0154 (4)	0.0016 (3)	0.0078 (3)	−0.0005 (3)
N2	0.0187 (4)	0.0205 (4)	0.0230 (4)	−0.0024 (3)	0.0110 (4)	−0.0036 (3)
C1	0.0228 (5)	0.0213 (5)	0.0217 (5)	−0.0031 (4)	0.0086 (4)	0.0007 (4)
C2	0.0208 (5)	0.0218 (5)	0.0182 (5)	0.0010 (4)	0.0038 (4)	−0.0003 (4)
C3	0.0253 (5)	0.0198 (5)	0.0204 (5)	0.0056 (4)	0.0085 (4)	−0.0010 (4)
C10	0.0179 (5)	0.0209 (5)	0.0215 (5)	−0.0007 (4)	0.0091 (4)	−0.0007 (4)
C4	0.0445 (7)	0.0246 (6)	0.0213 (5)	0.0068 (5)	0.0191 (5)	0.0022 (4)
C5	0.0260 (6)	0.0242 (6)	0.0222 (5)	0.0017 (5)	0.0030 (4)	0.0006 (4)
C11	0.0230 (5)	0.0187 (5)	0.0355 (6)	−0.0030 (4)	0.0140 (5)	−0.0027 (4)
C6	0.0331 (6)	0.0193 (5)	0.0239 (5)	−0.0043 (4)	0.0121 (5)	0.0011 (4)
C12	0.0297 (6)	0.0400 (7)	0.0269 (6)	−0.0018 (5)	0.0148 (5)	−0.0086 (5)

### Geometric parameters (Å, º)

**Table:**

Si1—N1	1.6878 (9)	C1—H1C	0.9800
Si1—C3	1.8825 (11)	C2—H2A	0.9800
Si1—C1	1.8871 (11)	C2—H2B	0.9800
Si1—C2	1.8990 (11)	C2—H2C	0.9800
Si1—Na1	3.3176 (5)	C3—H3A	0.9800
Si1—Na1^i^	3.3185 (5)	C3—H3B	0.9800
Si2—N1	1.6874 (9)	C3—H3C	0.9800
Si2—C4	1.8798 (11)	C10—C10^ii^	1.526 (2)
Si2—C5	1.8890 (12)	C10—H10A	0.9900
Si2—C6	1.8904 (12)	C10—H10B	0.9900
Si2—Na1^i^	3.3667 (7)	C4—H4A	0.9800
Si2—Na1	3.4814 (6)	C4—H4B	0.9800
Na1—N1^i^	2.4356 (10)	C4—H4C	0.9800
Na1—N1	2.4509 (9)	C5—H5A	0.9800
Na1—N2	2.5665 (10)	C5—H5B	0.9800
Na1—C2^i^	3.0415 (12)	C5—H5C	0.9800
Na1—Na1^i^	3.1548 (9)	C11—H11A	0.9800
Na1—Si1^i^	3.3185 (6)	C11—H11B	0.9800
Na1—Si2^i^	3.3667 (7)	C11—H11C	0.9800
N1—Na1^i^	2.4356 (10)	C6—H6A	0.9800
N2—C12	1.4718 (14)	C6—H6B	0.9800
N2—C11	1.4748 (14)	C6—H6C	0.9800
N2—C10	1.4767 (13)	C12—H12A	0.9800
C1—H1A	0.9800	C12—H12B	0.9800
C1—H1B	0.9800	C12—H12C	0.9800
			
N1—Si1—C3	118.58 (5)	Si1—N1—Na1^i^	105.75 (4)
N1—Si1—C1	112.81 (5)	Si2—N1—Na1	113.26 (4)
C3—Si1—C1	103.65 (5)	Si1—N1—Na1	105.07 (4)
N1—Si1—C2	109.73 (5)	Na1^i^—N1—Na1	80.42 (3)
C3—Si1—C2	105.54 (5)	C12—N2—C11	107.94 (9)
C1—Si1—C2	105.51 (5)	C12—N2—C10	108.31 (8)
N1—Si1—Na1	45.51 (3)	C11—N2—C10	110.27 (8)
C3—Si1—Na1	153.67 (4)	C12—N2—Na1	101.78 (6)
C1—Si1—Na1	73.46 (4)	C11—N2—Na1	109.96 (6)
C2—Si1—Na1	100.34 (4)	C10—N2—Na1	117.86 (6)
C3—Si1—Na1^i^	132.10 (4)	Si1—C1—H1A	109.5
C1—Si1—Na1^i^	124.25 (4)	Si1—C1—H1B	109.5
C2—Si1—Na1^i^	64.79 (3)	H1A—C1—H1B	109.5
Na1—Si1—Na1^i^	56.771 (14)	Si1—C1—H1C	109.5
N1—Si2—C4	116.24 (5)	H1A—C1—H1C	109.5
N1—Si2—C5	114.14 (5)	H1B—C1—H1C	109.5
C4—Si2—C5	104.49 (6)	Si1—C2—H2A	109.5
N1—Si2—C6	110.93 (5)	Si1—C2—H2B	109.5
C4—Si2—C6	105.31 (5)	H2A—C2—H2B	109.5
C5—Si2—C6	104.70 (5)	Si1—C2—H2C	109.5
C4—Si2—Na1^i^	128.00 (4)	H2A—C2—H2C	109.5
C5—Si2—Na1^i^	127.42 (4)	H2B—C2—H2C	109.5
C6—Si2—Na1^i^	67.51 (4)	Si1—C3—H3A	109.5
C4—Si2—Na1	149.32 (4)	Si1—C3—H3B	109.5
C5—Si2—Na1	79.30 (4)	H3A—C3—H3B	109.5
C6—Si2—Na1	102.97 (4)	Si1—C3—H3C	109.5
Na1^i^—Si2—Na1	54.832 (15)	H3A—C3—H3C	109.5
N1^i^—Na1—N1	99.58 (3)	H3B—C3—H3C	109.5
N1^i^—Na1—N2	129.70 (3)	N2—C10—C10^ii^	112.28 (11)
N1—Na1—N2	130.73 (3)	N2—C10—H10A	109.1
N1^i^—Na1—C2^i^	63.70 (3)	C10^ii^—C10—H10A	109.1
N1—Na1—C2^i^	106.50 (3)	N2—C10—H10B	109.1
N2—Na1—C2^i^	96.82 (3)	C10^ii^—C10—H10B	109.1
N1^i^—Na1—Na1^i^	50.00 (2)	H10A—C10—H10B	107.9
N1—Na1—Na1^i^	49.58 (2)	Si2—C4—H4A	109.5
N2—Na1—Na1^i^	179.70 (3)	Si2—C4—H4B	109.5
C2^i^—Na1—Na1^i^	83.03 (3)	H4A—C4—H4B	109.5
N1^i^—Na1—Si1	105.13 (3)	Si2—C4—H4C	109.5
N1—Na1—Si1	29.42 (2)	H4A—C4—H4C	109.5
N2—Na1—Si1	118.63 (3)	H4B—C4—H4C	109.5
C2^i^—Na1—Si1	135.23 (3)	Si2—C5—H5A	109.5
Na1^i^—Na1—Si1	61.629 (15)	Si2—C5—H5B	109.5
N1^i^—Na1—Si1^i^	29.31 (2)	H5A—C5—H5B	109.5
N1—Na1—Si1^i^	104.73 (2)	Si2—C5—H5C	109.5
N2—Na1—Si1^i^	118.14 (2)	H5A—C5—H5C	109.5
C2^i^—Na1—Si1^i^	34.40 (2)	H5B—C5—H5C	109.5
Na1^i^—Na1—Si1^i^	61.599 (14)	N2—C11—H11A	109.5
Si1—Na1—Si1^i^	123.228 (14)	N2—C11—H11B	109.5
N1^i^—Na1—Si2^i^	28.45 (2)	H11A—C11—H11B	109.5
N1—Na1—Si2^i^	108.56 (3)	N2—C11—H11C	109.5
N2—Na1—Si2^i^	115.30 (3)	H11A—C11—H11C	109.5
C2^i^—Na1—Si2^i^	87.66 (2)	H11B—C11—H11C	109.5
Na1^i^—Na1—Si2^i^	64.434 (19)	Si2—C6—H6A	109.5
Si1—Na1—Si2^i^	99.467 (17)	Si2—C6—H6B	109.5
Si1^i^—Na1—Si2^i^	54.922 (9)	H6A—C6—H6B	109.5
N1^i^—Na1—Si2	105.53 (2)	Si2—C6—H6C	109.5
N1—Na1—Si2	26.44 (2)	H6A—C6—H6C	109.5
N2—Na1—Si2	119.53 (3)	H6B—C6—H6C	109.5
C2^i^—Na1—Si2	85.96 (2)	N2—C12—H12A	109.5
Na1^i^—Na1—Si2	60.735 (17)	N2—C12—H12B	109.5
Si1—Na1—Si2	53.868 (11)	H12A—C12—H12B	109.5
Si1^i^—Na1—Si2	97.166 (12)	N2—C12—H12C	109.5
Si2^i^—Na1—Si2	125.168 (15)	H12A—C12—H12C	109.5
Si2—N1—Si1	131.98 (5)	H12B—C12—H12C	109.5
Si2—N1—Na1^i^	108.11 (4)		
			
N1—Si1—Na1—N1^i^	82.61 (5)	C6—Si2—Na1—Si2^i^	50.03 (4)
C3—Si1—Na1—N1^i^	144.37 (8)	Na1^i^—Si2—Na1—Si2^i^	0.0
C1—Si1—Na1—N1^i^	−128.21 (4)	C4—Si2—N1—Si1	14.19 (9)
C2—Si1—Na1—N1^i^	−24.95 (4)	C5—Si2—N1—Si1	−107.58 (8)
Na1^i^—Si1—Na1—N1^i^	25.47 (2)	C6—Si2—N1—Si1	134.44 (7)
C3—Si1—Na1—N1	61.76 (9)	Na1^i^—Si2—N1—Si1	132.73 (9)
C1—Si1—Na1—N1	149.18 (5)	Na1—Si2—N1—Si1	−140.13 (9)
C2—Si1—Na1—N1	−107.56 (5)	C4—Si2—N1—Na1^i^	−118.55 (6)
Na1^i^—Si1—Na1—N1	−57.14 (4)	C5—Si2—N1—Na1^i^	119.69 (5)
N1—Si1—Na1—N2	−123.05 (5)	C6—Si2—N1—Na1^i^	1.71 (6)
C3—Si1—Na1—N2	−61.29 (9)	Na1—Si2—N1—Na1^i^	87.14 (5)
C1—Si1—Na1—N2	26.13 (4)	C4—Si2—N1—Na1	154.32 (5)
C2—Si1—Na1—N2	129.40 (4)	C5—Si2—N1—Na1	32.55 (6)
Na1^i^—Si1—Na1—N2	179.82 (4)	C6—Si2—N1—Na1	−85.43 (6)
N1—Si1—Na1—C2^i^	14.89 (5)	Na1^i^—Si2—N1—Na1	−87.14 (5)
C3—Si1—Na1—C2^i^	76.65 (9)	C3—Si1—N1—Si2	−11.16 (9)
C1—Si1—Na1—C2^i^	164.07 (5)	C1—Si1—N1—Si2	110.22 (7)
C2—Si1—Na1—C2^i^	−92.66 (5)	C2—Si1—N1—Si2	−132.46 (7)
Na1^i^—Si1—Na1—C2^i^	−42.24 (3)	Na1—Si1—N1—Si2	142.42 (9)
N1—Si1—Na1—Na1^i^	57.14 (4)	Na1^i^—Si1—N1—Si2	−133.50 (9)
C3—Si1—Na1—Na1^i^	118.89 (8)	C3—Si1—N1—Na1^i^	122.33 (5)
C1—Si1—Na1—Na1^i^	−153.69 (4)	C1—Si1—N1—Na1^i^	−116.29 (5)
C2—Si1—Na1—Na1^i^	−50.42 (4)	C2—Si1—N1—Na1^i^	1.04 (5)
N1—Si1—Na1—Si1^i^	57.14 (4)	Na1—Si1—N1—Na1^i^	−84.09 (4)
C3—Si1—Na1—Si1^i^	118.89 (8)	C3—Si1—N1—Na1	−153.58 (4)
C1—Si1—Na1—Si1^i^	−153.69 (4)	C1—Si1—N1—Na1	−32.20 (6)
C2—Si1—Na1—Si1^i^	−50.42 (4)	C2—Si1—N1—Na1	85.13 (5)
Na1^i^—Si1—Na1—Si1^i^	0.0	Na1^i^—Si1—N1—Na1	84.09 (4)
N1—Si1—Na1—Si2^i^	111.17 (4)	N1^i^—Na1—N1—Si2	105.70 (5)
C3—Si1—Na1—Si2^i^	172.93 (8)	N2—Na1—N1—Si2	−74.29 (6)
C1—Si1—Na1—Si2^i^	−99.65 (4)	C2^i^—Na1—N1—Si2	40.45 (5)
C2—Si1—Na1—Si2^i^	3.61 (4)	Na1^i^—Na1—N1—Si2	105.70 (5)
Na1^i^—Si1—Na1—Si2^i^	54.035 (14)	Si1—Na1—N1—Si2	−150.43 (7)
N1—Si1—Na1—Si2	−15.79 (4)	Si1^i^—Na1—N1—Si2	76.17 (5)
C3—Si1—Na1—Si2	45.97 (8)	Si2^i^—Na1—N1—Si2	133.58 (4)
C1—Si1—Na1—Si2	133.39 (4)	N1^i^—Na1—N1—Si1	−103.87 (4)
C2—Si1—Na1—Si2	−123.35 (4)	N2—Na1—N1—Si1	76.14 (6)
Na1^i^—Si1—Na1—Si2	−72.925 (18)	C2^i^—Na1—N1—Si1	−169.12 (4)
N1—Si2—Na1—N1^i^	−80.15 (6)	Na1^i^—Na1—N1—Si1	−103.87 (4)
C4—Si2—Na1—N1^i^	−129.77 (9)	Si1^i^—Na1—N1—Si1	−133.41 (3)
C5—Si2—Na1—N1^i^	129.83 (4)	Si2^i^—Na1—N1—Si1	−75.99 (4)
C6—Si2—Na1—N1^i^	27.03 (5)	Si2—Na1—N1—Si1	150.43 (7)
Na1^i^—Si2—Na1—N1^i^	−23.00 (2)	N1^i^—Na1—N1—Na1^i^	0.0
C4—Si2—Na1—N1	−49.62 (9)	N2—Na1—N1—Na1^i^	−179.99 (4)
C5—Si2—Na1—N1	−150.02 (6)	C2^i^—Na1—N1—Na1^i^	−65.25 (3)
C6—Si2—Na1—N1	107.18 (6)	Si1—Na1—N1—Na1^i^	103.87 (4)
Na1^i^—Si2—Na1—N1	57.15 (5)	Si1^i^—Na1—N1—Na1^i^	−29.54 (2)
N1—Si2—Na1—N2	123.03 (6)	Si2^i^—Na1—N1—Na1^i^	27.88 (2)
C4—Si2—Na1—N2	73.41 (9)	Si2—Na1—N1—Na1^i^	−105.70 (5)
C5—Si2—Na1—N2	−26.99 (5)	N1^i^—Na1—N2—C12	−148.37 (7)
C6—Si2—Na1—N2	−129.79 (5)	N1—Na1—N2—C12	31.62 (9)
Na1^i^—Si2—Na1—N2	−179.82 (4)	C2^i^—Na1—N2—C12	−87.10 (7)
N1—Si2—Na1—C2^i^	−141.42 (5)	Si1—Na1—N2—C12	64.53 (7)
C4—Si2—Na1—C2^i^	168.96 (8)	Si1^i^—Na1—N2—C12	−115.64 (7)
C5—Si2—Na1—C2^i^	68.56 (4)	Si2^i^—Na1—N2—C12	−177.74 (6)
C6—Si2—Na1—C2^i^	−34.24 (4)	Si2—Na1—N2—C12	2.10 (8)
Na1^i^—Si2—Na1—C2^i^	−84.27 (3)	N1^i^—Na1—N2—C11	97.39 (7)
N1—Si2—Na1—Na1^i^	−57.15 (5)	N1—Na1—N2—C11	−82.62 (8)
C4—Si2—Na1—Na1^i^	−106.77 (8)	C2^i^—Na1—N2—C11	158.66 (7)
C5—Si2—Na1—Na1^i^	152.83 (4)	Si1—Na1—N2—C11	−49.71 (7)
C6—Si2—Na1—Na1^i^	50.03 (4)	Si1^i^—Na1—N2—C11	130.12 (6)
N1—Si2—Na1—Si1	17.47 (4)	Si2^i^—Na1—N2—C11	68.02 (7)
C4—Si2—Na1—Si1	−32.16 (8)	Si2—Na1—N2—C11	−112.14 (6)
C5—Si2—Na1—Si1	−132.56 (4)	N1^i^—Na1—N2—C10	−30.12 (9)
C6—Si2—Na1—Si1	124.65 (4)	N1—Na1—N2—C10	149.86 (7)
Na1^i^—Si2—Na1—Si1	74.616 (18)	C2^i^—Na1—N2—C10	31.14 (7)
N1—Si2—Na1—Si1^i^	−108.83 (5)	Si1—Na1—N2—C10	−177.22 (6)
C4—Si2—Na1—Si1^i^	−158.46 (8)	Si1^i^—Na1—N2—C10	2.61 (8)
C5—Si2—Na1—Si1^i^	101.14 (4)	Si2^i^—Na1—N2—C10	−59.49 (7)
C6—Si2—Na1—Si1^i^	−1.66 (4)	Si2—Na1—N2—C10	120.35 (6)
Na1^i^—Si2—Na1—Si1^i^	−51.686 (12)	C12—N2—C10—C10^ii^	173.03 (11)
N1—Si2—Na1—Si2^i^	−57.15 (5)	C11—N2—C10—C10^ii^	−69.05 (13)
C4—Si2—Na1—Si2^i^	−106.77 (8)	Na1—N2—C10—C10^ii^	58.31 (13)
C5—Si2—Na1—Si2^i^	152.83 (4)		

Symmetry codes: (i) −*x*+2, −*y*, −*z*; (ii) −*x*+3, −*y*, −*z*.
